# Immune mediators in heart–lung communication

**DOI:** 10.1007/s00424-024-03013-z

**Published:** 2024-09-11

**Authors:** Jonathan L. Gillan, Lara Jaeschke, Wolfgang M. Kuebler, Jana Grune

**Affiliations:** 1 Department of Cardiothoracic and Vascular Surgery, Deutsches Herzzentrum Der Charité (DHZC), Virchowweg 6, 10117 Berlin, Germany; 2 Institute of Physiology, Charité-Universitätsmedizin Berlin, Charitéplatz 1, 10117 Berlin, Germany; 3 DZHK (German Centre for Cardiovascular Research), Partner Site Berlin, Berlin, Germany

**Keywords:** Organ crosstalk, Interorgan communication, Cardiopulmonary axis, Heart–lung immunology, Pulmonary hypertension

## Abstract

It is often the case that serious, end-stage manifestations of disease result from secondary complications in organs distinct from the initial site of injury or infection. This is particularly true of diseases of the heart–lung axis, given the tight anatomical connections of the two organs within a common cavity in which they collectively orchestrate the two major, intertwined circulatory pathways. Immune cells and the soluble mediators they secrete serve as effective, and targetable, messengers of signals between different regions of the body but can also contribute to the spread of pathology. In this review, we discuss the immunological basis of interorgan communication between the heart and lung in various common diseases, and in the context of organ crosstalk more generally. Gaining a greater understanding of how the heart and lung communicate in health and disease, and viewing disease progression generally from a more holistic, whole-body viewpoint have the potential to inform new diagnostic approaches and strategies for better prevention and treatment of comorbidities.

## Organ crosstalk and its clinical relevance

Organs communicate with each other in order to maintain homeostasis of the organism in a steady state and to react to acute or chronic injury in disease. Many diseases ostensibly affect only one primary organ but are, in reality, multi-organ diseases with substantial remote end-organ damage. Secondary organ complications are often misinterpreted as less important to the disease outcome but often represent key drivers of morbidity and mortality in patients lacking therapeutic options. Our organ-centric view in physiology has been challenged in the past decades by previously unexpected coordinated interorgan crosstalk regulated by soluble circulating molecules, the immune system, and neuronal signals [53]. This cumulative evidence has led to a deeper understanding of complex diseases affecting one tissue predominantly but are largely caused by disruption of regulatory signals outside of this central site. How exactly an acute insult in one organ can drive secondary injury in another, and the clinical importance of this in several common diseases remains poorly understood. In this review, we will detail interorgan communication of the heart and lung via immunomodulatory circuits and how this particular mode of crosstalk helps to shape outcomes in diseases of the cardiopulmonary axis. We will begin by introducing established heart-organ and lung-organ axes in the context of inflammation and their clinical relevance. We will then focus on cardiopulmonary communication in the steady state and discuss immune system-mediated heart–lung communication in diseases such as pulmonary hypertension, acute respiratory distress syndrome (ARDS), and pneumonia. The repertoire of organs communicating with each other and mechanisms involved in interorgan communication outlined in this review is by no means complete.

### The heart, lungs, and peripheral organs

Interorgan communication via inflammatory signaling involving the heart has been described by a multitude of studies, including the description of a heart-spleen axis driving disease progression after myocardial infarction by macrophage-mediated extramedullary monocytopoiesis in the spleen [26, 27], a heart-adipose tissue axis where adipose tissue contributes to systemic inflammation which exacerbates cardiac remodelling in heart failure (HF) [3, 80, 114], a heart-skeletal muscle axis associated with skeletal muscle wasting in heart failure patients, perhaps mediated by tumour necrosis factor (TNF)-α secretion from the failing heart [28, 60, 117], and even the existence of a heart-bone axis has been suggested, connecting myocardial infarction and osteoporotic fractures [112]. Specifically, activation of the nucleotide-binding oligomerization domain-like receptors with pyrin domain containing 3 (NLRP3) inflammasome is likely to have a central role in cardiac remodelling and mediating crosstalk with peripheral organs [48]. Upon cardiac injury, NLRP3 inflammasome activation, primarily in local macrophages as well as other recruited myeloid cells, causes the downstream production and secretion of inflammatory cytokines interleukin (IL)-1β and IL-18. It has been shown that expression of IL-18 in the myocardium, as well as in the circulation, increases significantly and in tandem in patients with HF, particularly in those who die from the condition [65]. Another study demonstrated that intravenous administration of serum from HF patients into wild-type mice induced left ventricular (LV) systolic dysfunction but concomitant addition of an inhibitor of IL-18, IL-18-binding protein (IL-18BP), was sufficient to prevent the development of cardiac dysfunction, thereby demonstrating that circulating cytokines derived from immune cells in the HF-damaged heart, can act as a carrier of inflammation between tissues [113].

### The heart-brain axis

Neurocardiology has been conceptualized into three major categories: the heart’s effect on the brain (e.g. cardiac source for embolic strokes), the brain’s effect on the heart (e.g. stroke causes cardiac effects), and neurocardiac syndromes (e.g. Friedreich disease) [48]. Moving from the heart to the brain, the powerful systemic inflammatory response arising post-myocardial infarction is initiated by the release of inflammatory cytokines and chemokines by local myeloid cells, endothelial cells, and fibroblasts reacting to danger signals released from necrotic cardiomyocytes [85]. In turn, this leads to increased monocyte infiltration and expansion of microglia and resident perivascular macrophage populations within the brain [111]. In this context, monocytes serve as conduits of inflammation between the heart and the brain, interacting with oligodendrocytes in order to exacerbate neuroinflammation and drive white matter injury, with the aforementioned study also showing that blocking the ability of monocytes to infiltrate tissue with *Ccr2*.^−/−^ mice prevents white matter injury and preserves cognitive function post-myocardial infarction (MI). Positron emission tomography (PET) imaging of the mitochondrial translocator protein (TSPO), used as a marker of activated immune cells, revealed activation of cerebral microglia to be the hallmark of post-MI neuroinflammation, with a heightened signal throughout the cortex but primarily located in the temporal and frontobasal cortex, as well as the hypothalamus and cerebellum, post-MI in both mice and humans [110]. This interaction potentially also contributes to the cognitive dysfunction associated with the development of vascular dementia, the incidence of which is significantly higher post-MI [106].

In the other direction, stroke and cardiac diseases are among the world’s leading causes of morbidity and mortality and new-onset cardiovascular events following ischemic stroke are common and associated with worse prognosis, known as the ‘stroke-heart syndrome’ (SHS) [16, 38, 94, 97]. Within 30 days after ischemic stroke, approximately 9% of stroke patients develop atrial fibrillation (AF) and 1.2% of life-threatening ventricular tachycardia (VT), leading to sudden cardiac death in a relevant number of patients [16, 97]. Long-term stroke survivors remain at high risk of incident major adverse cardiovascular events (MACE) and HF [41]. This risk of incident MACE is 1.6- to 2.0-fold higher in patients with early post-stroke cardiac complications compared to those without [44]. Experimental stroke models in mice have been shown to lead to drastic alterations in the composition and function of splenic macrophage populations and thus may also impact cardiac function via the aforementioned heart-spleen axis [69, 70]. One recent study demonstrated the occurrence of trained immunity, persisting epigenetic changes in innate immune cells in response to a primary stimulus that confers non-specific immune memory and a heightened response to a secondary stimulus, in myeloid cells following stroke [99]. An experimental stroke model led to chronic cardiac diastolic dysfunction and cardiac fibrosis associated with heightened cardiac inflammation, particularly due to a drastic increase in monocyte recruitment to the heart. Single-cell RNA sequencing (scRNAseq) revealed long-term transcriptional changes to monocytes and macrophages in the blood and tissues, the majority of which were associated with a pro-inflammatory phenotype, at 1-month post-stroke and also uncovered distinct post-stroke transcriptional signatures of haematopoietic stem and progenitor cells (HSPCs) in the bone marrow, a hallmark of trained immunity. Transcriptional changes to myeloid cells and increased recruitment of inflammatory monocytes were particularly prominent in the heart and, accordingly, both post-stroke chronic inflammation and cardiac dysfunction were transmissible by HSPC-enriched bone marrow (BM) transfer from 1-month post-stroke mice to naïve recipients. Single-cell assay for transposase-accessible chromatin with sequencing (ATAC-seq) of the BM revealed IL-1β, known to be elevated systemically after stroke, to be a key driver of epigenetic reprogramming in HSPCs post-stroke. Subsequently, both administrations of IL-1β-neutralising antibodies or the CCR2/CCR5 dual antagonist, cenicriviroc, to block migration of myeloid cells, was sufficient to substantially reduce infiltration of monocytes into the heart, reduce cardiac fibrosis and prevent cardiac dysfunction post-stroke. This illustrates the therapeutic potential of targeting immune mediators as a bridge between primary disease and secondary comorbidities.

### The heart-kidney axis

Renal dysfunction is strongly associated with disease progression and clinical outcome in patients with HF. Mortality worsens incrementally across the range of renal function in HF patients evident as a two-fold increased risk of mortality in HF patients with renal impairment compared to patients with normal renal function [102]. Additionally, kidney injury often develops in patients with acute cardiac illness such as acute decompensated HF [93], suggesting a strongly intertwined crosstalk of the kidneys and the heart in HF pathophysiology [9]. As demonstrated in one elegant mechanistic study, the complex interorgan network involving the heart and kidneys is essential for the adaptive response to pressure overload of the left ventricle [34]. The authors showed that blocking the accumulation of inflammatory monocytes and macrophages in the kidney with collecting duct (CD) cell-specific deletion of the transcription factor, Klf4 (CD-*Klf5*KO), led to a failed myocardial adaptive response to chronic pressure overload, induced by transverse aortic constriction (TAC), and subsequent development of HF. Following this pressure afterload, numbers of Ly6C^lo^ macrophages were selectively increased in the heart, where they persisted for at least 28 days post-TAC but, crucially, this increase was suppressed in CD-*Klf5*KO mice, thereby highlighting the ability of the kidney to affect the immune cell profile of the heart. Gene analysis of the kidney and blood revealed colony stimulating factor 2 (CSF2) to be a potential intermediary between the organs given that its expression is drastically increased in the serum and kidney post-TAC but significantly less so in CD-*Klf5*KO mice. Indeed, administration via osmotic mini-pump of an anti-CSF2 neutralising antibody 3 days pre-TAC completely blocked the TAC-induced increase in cardiac Ly6C^lo^ macrophages seen in WT mice. The model proposes that in response to pressure overload, resident renal macrophages produce TNF-α, which in turn activates renal endothelial cells (ECs) to secrete CSF2 into the circulation. Within the heart, CSF2 activates cardiac Ly6C^lo^ macrophages, contributing to the heart’s adaptive response to pressure overload by producing amphiregulin (AREG) and thereby activating the cardiac hypertrophic program [34]. This intricate example of interorgan crosstalk demonstrates that peripheral organs are capable of controlling local proliferation of resident cell populations in remote injured organs.

### The lung-kidney axis

Multi-organ failure involving the kidney–lung axis is an often fatal manifestation of bidirectional interorgan communication that is frequently seen in the intensive care unit (ICU) [14, 63]. Patients with viral or bacterial lung infections leading to the development of ARDS, frequently co-develop acute kidney injury associated with increased mortality, for reasons that are incompletely understood [7, 86]. In line with this, up to 70% of patients with lung transplants develop acute kidney injury within 24 h of engraftment, significantly increasing their 5-year mortality rate [58, 98]. In one study, ligand-receptor (L-R) pairing analysis, which uses transcriptomics data to characterise specific molecules that mediate cell–cell communication, was performed on gene expression data from lung and kidney single-cell RNA sequencing (scRNAseq) datasets, acquired both at baseline and following acute kidney injury (AKI)–induced acute lung injury (ALI) [50]. This was carried out using the machine learning algorithm, CellPhoneDB, which consists of an enormous repository of ligands, receptors, and specific interactions between the two [29]. It was shown that kidney tubule-derived osteopontin (OPN) and CD44 in the lung demonstrated the strongest interaction of all ligand-receptor pairings both at baseline and after kidney injury. Inhibition of kidney-derived OPN, either using a global OPN KO mouse or by administering an anti-OPN neutralizing antibody, prevented AKI-ALI and respiratory failure, whilst AKI-ALI could be induced in WT mice upon transplantation of kidneys from ischemic WT, but not ischemic OPN KO mice. This demonstrates that the release of a kidney-derived mediator into the circulation is sufficient to spread and evolve disease in the kidney into a remote, secondary organ. Whether or not the kidney can act as a direct source of inflammatory mediators such as cytokines which spread inflammation to secondary organs is less clear but both the aforementioned study and others have indicated that neutrophil and macrophage production of IL-1β and IL-6, respectively, in the injured kidney results in subsequent release of these cytokines into circulation [52]. The prevalence of pulmonary hypertension (PH) in patients with chronic kidney disease (CKD) was shown in one study to be increased two to eight-fold, compared to the general population [13]. In the same study, 81% of CKD patients undergoing haemodialysis treatment developed PH, which may suggest common pathophysiology in kidney and lung injury.

The clinical evidence for secondary organ damage in disease is clear yet the precise mechanisms that aid communication between organs in health and disease are less well known. Anatomically speaking, the organ axes outlined above are examples of communication between two distal sites. Given the unique anatomical and physiological coupling of the heart and lung, the cardiopulmonary axis serves as a particularly efficient conduit for the movement of biological signals.

## Mechanisms of communication between the heart and lung

### Basic interorgan communication networks

The most basic and effective way through which body systems communicate during homeostasis is via the endocrine and autonomic nervous systems. Insulin and glucagon, secreted following glucose sensing by beta cells of the pancreas, act primarily upon the liver to regulate blood sugar levels via glycogenesis and glycogenolysis, respectively. Insulin, along with adipose tissue-derived leptin, stomach-derived ghrelin, cholecystokinin (CCK), and others, also communicates directly with the brain through activation of local neuronal pathways that transmit signals to nerve centres in the hypothalamus to regulate food intake via stimulation or suppression of appetite [54]. The renin–angiotensin–aldosterone system (RAAS) is a crucial multi-organ neurohormonal system, the core function of which is to control blood volume through regulation of salt/water homeostasis in the kidneys [96]. Juxtaglomerular cells of the kidneys secrete renin which acts to cleave liver-derived angiotensinogen. Angiotensinogen converting enzyme (ACE) is expressed primarily on endothelial cells in the lung and catalyses the conversion of angiotensin I to angiotensin II, the latter of which can then induce the release of multifunctional mineralocorticoid, aldosterone, in the adrenal gland [81]. A greater depth of understanding of the various elements and organ interplay involved in RAAS has revealed further avenues for potential clinical intervention to target the RAAS cascade, particularly in the context of hyperaldosteronism, primary hypertension, as well as heart and kidney failure [92]. Besides active transmission of information via the neurohormonal system, there are also more passive mediators that aid heart–lung communication. Extracellular vesicles (EVs), including microvesicles, exosomes, and apoptotic bodies, can be secreted and taken up by virtually every cell in the body, transferring in the process a wide array of potential DNA/RNA-, lipid-, and protein-based cargo that can elicit potent biological effects upon uptake and surface marker binding by target cells, thus acting as rapid, soluble mediators of cell–cell communication [36, 61]. The flow of information in the form of inflammatory cytokines, microRNAs (miRNAs), and other mediators via EVs between cardiomyocytes, heart endothelial cells, smooth muscle cells, fibroblasts, and virtually all cardiac cells is central to the effective maintenance of heart homeostasis. The use of transgenic mice allowing inducible, cardiomyocyte-specific expression of green fluorescent protein (GFP) demonstrated the presence in the circulation of heart-derived EVs, even during homeostasis, with a significant increase in circulating myocardial EVs found in response to lipopolysaccharide (LPS)-induced systemic inflammation (SIRS), and consequent cardiac injury [43]. Others have found that EVs containing myocardial microRNAs released into the circulation following myocardial infarction can act directly upon remote tissues, including adipose tissue and bone marrow, with one study showing that EVs derived MI-mice and administered to WT mice accumulate in mononuclear cells of the bone marrow, causing downregulation of their expression of CXCR4, and a subsequent increase in mobilisation these cells into the circulation [19, 37]. In this context, EVs likely serve as a communicator of cardiac damage to the BM in order to mobilise progenitor cells and a pro-repair response but this remains ill-defined. Given that it has also been shown that the lung is the predominant end site of accumulation of circulating EVs, further investigation is justified into the role of myocardial-derived EVs in contributing to pulmonary complications secondary to heart disease [108]. The aforementioned study by Fujiu et al., in which they identify a heart-kidney axis orchestrating the adaptation of the heart to cardiac pressure overload via the stimulation of Ly6C^lo^ cardiac macrophages, is an example of an interorgan communication network mediated by neuroimmune connections. Such neuroimmune circuits refer to the bidirectional movement of environmental cues and stimuli between neuronal and immune cells and are crucial in mediating homeostasis but may also serve an underappreciated role in pathology [46]. Cardiac pressure overload in the left ventricle is sensed by afferent sympathetic nerves in the heart which innervate the kidneys and trigger the activation of renal CD cells and their subsequent release of S100A8–S100A9 calcium-binding proteins. S100A8–S100A9 then triggers the recruitment and pro-inflammatory differentiation of TNF-secreting macrophages which stimulate the release of CSF2 from renal endothelial cells. As such, renal sympathetic nerve ablation (RSNA) led to a decrease in serum levels of CSF2 and numbers of Ly6C^lo^ macrophages in the heart and consequently reduced LV systolic function and survival in TAC-operated mice [34]. The heart and lung neuronal labelling with neurotracers revealed the extensive presence of shared sensory neurons converging in the peripheral vagal ganglia, allowing coordinated regulation of overall cardiopulmonary function and cross-sensitisation of the two organs [24].

### The cardiopulmonary immune system

**Fig. 1:**
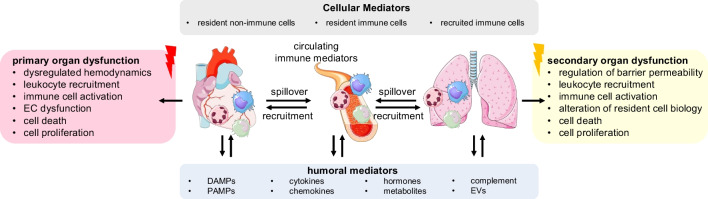
Mediators of interorgan communication between the heart and lung. Close anatomical proximity and extensive pulmonary vasculature permit bidirectional movement of an array of biological signals between the heart and lung in homeostasis and in disease. Cellular and soluble immune mediators are the key couriers of information between the two organs but also function as vehicles for the expansion of disease from the site of injury or infection. EC, endothelial cell; DAMPs, damage-associated molecular patterns; PAMPs, pathogen-associate molecular patterns; EVs, extracellular vesicles

Hormones and EVs are far from the only endocrine messengers that function to bring about transmission of information between distinct tissue sites (Fig. 1). Although the vast majority of tissues harbour resident immune defences, these alone would not be sufficient to effectively combat pathogenic invaders, or indeed bring about required tissue repair following pathogen clearance. This is demonstrated by leukocyte adhesion deficiencies (LADs), a rare class of inherited immunodeficiencies that hinder the ability of circulating immune cells, particularly neutrophils and monocytes, to adhere to the endothelium and extravasate to the tissue. The result of this deficiency is widespread, recurrent infection throughout life and defective wound healing, similar to the loss of effective lung repair and regeneration seen following partial pneumonectomy in mice deficient for the key monocyte migratory chemokine, C–C chemokine receptor type 2 (CCR2) [42, 56]. Simply put, an effective, controlled, and resolving immune response functions as one concerted, organ-spanning effort. The mammalian immune system, therefore, comprises a vast series of cellular and soluble mediators and, following local recognition of insult or injury, its core function is the engagement of targeted host defence and repair via the influx of immune mediators to said site, from distal regions. Integrated organ immunity functions not only to combat a specific pathogenic threat but also acts to prime the body’s wider immune defence as a whole to subsequent infectious stimuli, even those caused by a different pathogen, in a site that is completely remote from the initial immune stimulus, in a so-called ‘antigen-agnostic’ manner [87]. An effective cell-mediated immune response will be highly targeted and confined to the area of insult. However, in the context of disease, non-resolving inflammatory responses can grow to exceed the scale of the initial stimulus and, in turn, can exert off-target, collateral damage. Given that the heart and lung share tightly connected anatomical structures and large circulatory systems, here we explore what is known of the spillover of disease across this axis.

### Lung inflammation as the risk factor for cardiovascular diseases

The anatomical coupling of the lung and heart and the fact that the lung has the greatest vascular density of any human organ provide a unique passageway for the rapid and extensive bidirectional movement of cellular and soluble immune mediators between the two. Thus, inflammation in one tissue is particularly and intrinsically linked to a concomitant inflammatory phenotype in the other. Inhalation of small particulate matter, including in the form of cigarette smoke but also more generally in polluted industrial emissions, is a major contributor to lung injury and ultimately to chronic inflammation in the lower airways [119]. Macrophage-derived procoagulant inflammatory cytokine, IL-6, is known to translocate from the lung to the circulation in response to ambient particulate matter in much the same way as occurs following intrathecal exposure of mice to LPS, contributing to systemic inflammation and vascular dysfunction, as assessed by measuring vasorelaxation responses of abdominal aorta rings to acetylcholine (ACh) [51, 109]. Given the well-described link between systemic inflammation and worsening cardiovascular disease, the spillover of such inflammatory mediators into the pulmonary vasculature potentially represents a key trigger for progression of cardiovascular disease and, specifically, the growth and eventual rupture of atherosclerotic plaques [115, 120]. One set of experiments performed in atherosclerosis-prone apolipoprotein E^−/−^ (ApoE^−/−^) mice who were exposed to particulate matter-containing aerosols demonstrated that inflammation, in particular the substantial infiltration of reactive oxygen species (ROS)-producing macrophages into the arterial plaque, showed a stronger correlation with plaque development than did levels of serum cholesterol in response to small particle exposure of the airways [105]. Inhalation of such particulate matter also induces a prothrombotic phenotype in mouse lungs whilst depletion of airway alveolar macrophages, by intrathecal administration of clodronate-liposomes, was shown to be sufficient to reverse thrombosis in these mice, by reducing levels of IL-6 [77]. Lung and systemic inflammation resulting from respiratory infections are also strongly linked with consequent cardiovascular complications. Influenza infection is associated with a significantly increased risk of acute cardiac events, including myocardial infarction, particularly during the first 3 days of infection [101]. Potential mechanisms to explain this association include direct infection of the cardiac tissue itself, helped by the ability of the virus to induce upregulation of ectopic trypsin in the heart which facilitates viral entry into host cells and subsequent myocarditis [79], as well as the increased risk of HF that exists following influenza-associated damage to the pulmonary architecture leading to lung vascular maladaptation. Local and systemic inflammation is another potential key factor linking acute respiratory infections, such as influenza, with acute coronary syndromes, particularly in individuals prone to cardiovascular complications such as those with atherosclerosis [22]. One study showed that infection of ApoE^−/−^ mice with influenza A led to acute inflammation and substantial infiltration of immune cells, particularly macrophages and lymphocytes, into aortic atherosclerotic plaques [78]. Accordingly, a meta-analysis study indicated that influenza vaccination is associated with a 34% reduction in the rate of major cardiovascular events rising to a 45% reduction in patients with acute coronary syndrome (ACS) [5, 84]. Similar secondary cardiovascular complications are of course seen in numerous infections related to systemic inflammatory responses, even in the case of individuals infected with human immunodeficiency virus (HIV) who have a dramatically increased risk of developing pulmonary arterial hypertension (PAH) (0.5% of HIV sufferers—approximately 100–1000 times higher rate than in non-HIV individuals) [72]. Interestingly, the development of PAH in these patients is closely associated with an increase in lung and systemic viral inflammation, independently of the presence of other concurrent HIV-associated lung manifestations such as hypoxemia or chronic obstructive pulmonary disease (COPD) [18, 74]. As such, levels of sputum neutrophils and IL-8 significantly positively correlate with pulmonary arterial pressure, as defined by tricuspid regurgitant jet velocity (TRV) and pulmonary artery systolic pressure (PASP), in individuals with HIV [39, 75]. Nevertheless, despite the potential for infection localised anywhere in the body to disturb heart homeostasis, respiratory infections are especially harmful in this context, as is also evident from the immensely heightened risk of numerous cardiovascular complications, including hypertension, dysrhythmias, and heart failure, secondary to infection with severe acute respiratory syndrome coronavirus 2 (SARS–CoV–2), both in the acute and convalescence stages of infection [67, 122]. The aforementioned trained immunity of innate immune cells is particularly well described in the context of acute infection in which immune training exists to protect against re-infection, but this heightened activation state in circulating monocytes in particular displays substantial transcriptional overlap with a pro-atherogenic phenotype and such it has been postulated that infection-trained monocytes may therefore contribute to the acceleration of plaque formation in individuals prone to atherosclerosis [57].

### Secondary pulmonary complications in disease of the heart

In the other direction, infections of the heart, such as infective endocarditis which is associated with severe infection and life-threatening inflammation of the endocardium (typically one or more of the heart valves), are commonly associated with secondary pulmonary complications and subsequent poor clinical outcome [17]. Right-sided endocarditis related to injection drug use can lead to embolization of infectious vegetation which is then propelled directly into the pulmonary circulation leading to an increased downstream risk of bacterial pneumonia, lung abscesses, and empynema [17]. Of course, infections originating in the heart are rare, especially relative to the airways, but immune responses stemming from injury to the heart more generally, particularly from damage associated with myocardial infarction (MI) and ischemia/reperfusion (I/R) injury, are far more common and of immense importance to clinical outcome in such patients [48]. Injury to the myocardium caused in these contexts stimulates the release of danger signals from damaged and dead cells, so-called damage-associated molecular patterns (DAMPs), including mitochondrial DNA (mtDNA), adenosine triphosphate (ATP), and high mobility group box-1 (HMGB1), which function much like pathogenic microbial products to elicit an inflammatory response. Sterile inflammation of this sort includes initial sensing of DAMPs by stromal cells and resident macrophages surrounding the coronary artery and in the pericardial space, and their consequent release of inflammatory cytokines and chemokines which, alongside degranulation of mast cells, function to activate the local endothelium and mobilise the rapid recruitment of primarily neutrophils and monocytes en masse [31, 107]. The engagement of a robust but balanced inflammatory response to MI, for example, is fundamentally protective to the host and is crucial for effective infarct healing and cardiac remodelling. However, prolonged, dysregulated immune responses to cardiac injury are common and can lead to adverse remodelling, systemic inflammation, and knock-on pulmonary complications [66]. A key aspect of an effective wound healing response post-MI is the orchestration of fibroblast recruitment and differentiation to collagen-producing myofibroblasts by local macrophage populations, primarily by their secretion of cytokines and growth factors, including transforming growth factor (TGF)-β and platelet-derived growth factor (PDGF) [121]. Connective tissue growth factor (CTGF) is another important profibrotic growth factor in this context and it has been shown that 7 days post-MI, levels of CTGF in the lungs increase significantly with remodelling of the pulmonary arteries, and localise predominantly to alveolar macrophages and interstitial fibroblasts, with alveolar macrophages in particular governing the spread of fibrosis from the heart to the lung vasculature and increasing the risk of pulmonary hypertension [1].

## Immune-mediated heart–lung communication in disease

The networks which allow for the movement of information between organs in homeostasis serve also to transport immune cells and pathogen- and injury-associated inflammatory mediators from sites of primary disease to secondary tissues, carrying with them the threat of secondary pathology. Regarding the heart–lung axis, this manifests as some of the most clinically burdensome comorbidities in the world. In this section, we outline some of the most relevant examples of this, including heart diseases (heart failure) associated with secondary pulmonary manifestations, as well as lung diseases (COPD and pneumonia) which commonly precede heightened risk of cardiovascular complications.

### Pulmonary hypertension

**Fig. 2:**
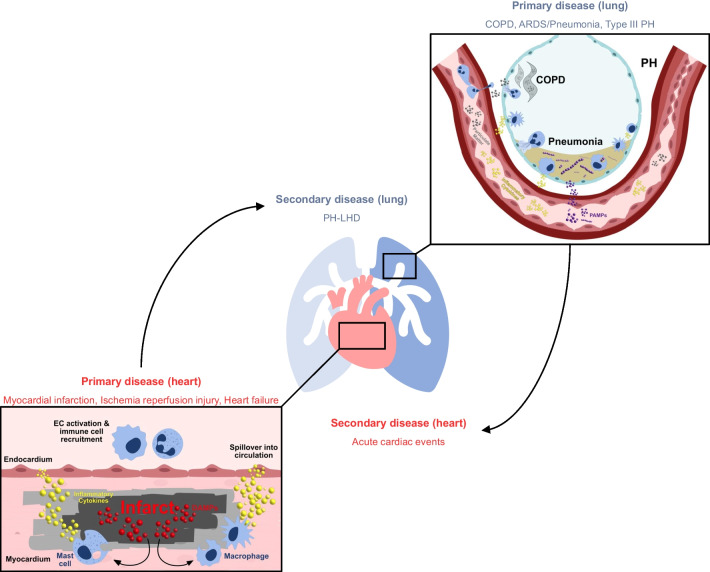
Proposed expansion of disease via immune mediators between the heart and lung. Infection and injury in the heart (myocardial infarction shown) or lung (COPD, pneumonia, and PH shown) can lead to the spillover of pathogen- and damage-associated immune mediators into circulation. The close proximity of these organs and the extensive nature of the pulmonary vasculature leads to a heightened risk of collateral damage in the other organ, and secondary disease associated with the bidirectional of these mediators. DAMPs, damage-associated molecular patterns; PAMPs, pathogen-associated molecular patterns; EC, endothelial cell; PH-LHD, pulmonary hypertension due to left heart disease; ARDS, acute respiratory distress syndrome; COPD, chronic obstructive pulmonary disease

The interplay between the heart and lung is a central facet of numerous disease progressions but none more so than in the context of PH. PH, defined by a resting mean pulmonary artery pressure (mPAP) ≥ 20 mmHg, is a common disorder frequently associated with various conditions on both sides of the cardiopulmonary axis, predominantly left-sided heart failure and lung disease [45, 47]. PH is divided into five subgroups: pulmonary arterial hypertension (PAH aka type I), pulmonary hypertension due to left-sided heart disease (PH-LHD aka type II), pulmonary hypertension due to lung disease or hypoxia (aka type III), chronic thromboembolic pulmonary hypertension (aka type IV), and pulmonary hypertension with unclear or multifactorial mechanisms (aka type V) [73]. The different classes of PH demonstrate clearly how the disease can be bidirectional in origin, either by originating in the heart (both the right and left side) and progressing quickly to the airways in the case of PH-LHD (type II), or vice versa whereby chronic inflammatory lung diseases (including COPD, interstitial lung disease, and high-altitude disorders) lead to pathogenic remodelling in the pulmonary vasculature and consequently put immense strain on the heart, collectively referred to as type III PH (Fig. 2). PH-LHD is the most common form of PH, accounting for 65–80% of all patients. In response to left ventricular pressure overload, end-stage HF patients develop retrograde increases in pulmonary venous pressure, lung fibrosis, and vascular remodelling, ultimately leading to RV hypertrophy and failure [35]. There are currently no effective treatments specifically for PH-LHD, despite its prevalence, due primarily to significant gaps in our understanding of the underlying mechanisms of the pathology and the effects of related comorbidities. Whilst increased congestion no doubt plays a major role in causing remodelling of the pulmonary vasculature in response to left-sided filling pressures, passive congestion is not sufficient by itself to explain the development of PH-LHD. A large community-based study of individuals with HFpEF showed that pulmonary artery systolic pressures remained higher in HFpEF patients compared to individuals with hypertension but no HF, even after adjusting for changes in pulmonary capillary wedge pressure. This, therefore, suggests the existence of secondary factors driving active remodelling of the pulmonary vascular in PH-LHD, produced by pre-capillary mechanisms, related to left heart disease [55]. Likewise, with regard to the heightened risk of right-sided HF resulting from pulmonary vascular resistance in PH-LHD, an increase in RV afterload alone is also not sufficient to explain RV dysfunction in PH [12]. Taken together, these studies indicate the presence of active, bidirectional mechanisms that both drive the progression of PH secondary to HF and also the development of RV dysfunction secondary to pulmonary vasculature maladaptation. Candidates for what those mechanisms may be include dysregulated neuroendocrine axis, which responds to a drop in tissue perfusion by releasing neurohormones that can drive hypertrophic remodelling of the RV (e.g. angiotensin II and endothelin-I), but also immune mediators originating from the dysfunctional left heart (including post-MI as described in the “Organ crosstalk and its clinical relevance” and “Mechanisms of communication between the heart and lung” sections and the pulmonary vasculature [11]. It is well established that left-sided HF and associated PH are propagated by a broad, intense inflammatory response that feeds into a viscous cycle of inflammation, endothelial dysfunction, and pulmonary vascular maladaptation [35]. Histological analysis of the pulmonary vasculature in explanted tissue from patients PH patients and animal models reveals mass accumulation of immune cells in and around the remodelled PH pulmonary vasculature and even the generation of ectopic lymphoid follicles adjacent to remodelled vessels, which provide a local source of adaptive immune responses that likely contribute to the non-resolving nature of PH-associated vascular inflammation [32, 83, 89, 116]. The inflamed milieu of the vessel wall in PH extends into the circulation, with heightened levels in the serum of numerous cytokines including TNF-α, IL-1β, IL-6, IL-8, and several inflammatory chemokines that have been shown to correlate with RV dysfunction, in PH patients [103, 123]. TNF-α and IL-6 are both known to be chronically elevated in the serum of HF patients and mice overexpressing TNF-α or IL-6 develop severe PH and hypertrophy in the RV [2, 33, 59, 104]. This includes the SP-C/TNF-α transgenic mouse, in which TNF-α is overexpressed in type II alveolar epithelial cells specifically, thus demonstrating a direct link between immune-mediated disease in the lung and cardiac dysfunction. Despite the inflammatory and immune profiles of PH being described in great depth, less is known on the specific involvement of inflammation in the transitions between LHD and PH, and between PH and RV failure. Given the clear clinical correlation and substantial overlap of immune mediators between the remodelled pulmonary vascular and the failing heart, there is a strong indication that inflammatory crosstalk between the heart and lung contributes to disease progression in PH, warranting further investigation, particularly with a view to addressing the lack of specific treatment for PH-LHD.

### COPD

Secondary heart complications are commonplace in patients with COPD, even in the absence of pulmonary hypertension. COPD is by some considerable distance the most prevalent non-infectious lung disease in the world, affecting an estimated 10% of the adult population equating to well over 450 million, and with smoking status representing the major risk factor [10]. Two separate studies of exacerbating and stable COPD, respectively, have corroborated the estimation that approximately 20% of individuals with COPD have unrecognised left-sided heart failure due, in part, to the obscuring of symptoms of one disease by symptoms of the other which altogether exhibit substantial overlap, including dyspnea and nocturnal cough [64, 95]. Even a significantly more conservative approximation would still equate to tens of millions of patients if these findings were to translate to a global scale. A higher risk of mortality exists for patients with concurrent COPD and cardiovascular disease as does an increased rate of hospitalisation for both diseases [71]. The major hallmarks of COPD are airflow obstruction and chronic airway inflammation, both of which feed directly into the increased risk of cardiovascular disease. Lung inflammation is evident from analysis of sputum and bronchoalveolar lavage fluid (BALF) which reveals widespread neutrophilia in COPD, including in the BALF which typically houses a leukocyte composition of 85–95% alveolar macrophages in the healthy lung. Macrophage numbers also increase in the COPD airways, by up to 20-fold in severe disease, but their overall phagocytic function is dramatically reduced due most likely to the effects of cigarette smoke and subsequent oxidative stress that this introduces to the airspace [6, 90]. Nevertheless, it is activated neutrophils that become the predominant immune cell type in the airways as the disease progresses and, in concert with recruited monocyte-derived macrophages, bring with them an increasingly potent inflammatory milieu of cytokines and proteolytic enzymes including neutrophil elastase (NE), TNF-α, IL-6, IL-8, IL-17, and many others, ultimately leading to spill over of said milieu into circulation and systemic inflammation [8]. Signs of systemic inflammation are present in the circulation of COPD patients with mild to severe airflow obstruction, including elevated leukocyte counts and levels of C-reactive protein (CRP) relative to individuals with no airway obstruction [100]. Higher serum CRP combined with severe airway obstruction was found to significantly increase the risk of cardiac injury compared to patients with severe obstruction/low CRP and no obstruction/high CRP, thus indicating a COPD/inflammatory interplay as a causative factor in driving secondary cardiac complications. As this inflammation progresses to a chronic state, myeloid cells activate a strong adaptive immune response with the proliferation of inflammatory Th1 and Th17 cells in particular and a concomitant drop in the numbers of protective, anti-inflammatory Tregs, altogether acting to further exacerbate the inflammatory response. Patients with both COPD and cardiovascular diseases (CVDs) exhibit higher circulating levels of IL-6, IL-8, and fibrinogen than individuals with only COPD [71]. This inflammatory phenotype stems in part from the damage incurred by inhaled cigarette smoke and the subsequent release of immune-activating DAMPs, as described in the previous section, but COPD inflammation is at its most pronounced during exacerbation which is typically triggered by bacterial and viral respiratory infections [82]. Such infections are particularly prevalent due, in no small part, to the aforementioned impaired microbicidal capabilities of COPD alveolar macrophages. Such spikes in lung and systemic inflammation are associated with increased incidence of cardiovascular events and indeed the risk of myocardial infarction or stroke in COPD is more than doubled within 1–5 days following exacerbation [25]. Whilst COPD can lead to EC dysfunction in the pulmonary vascular bed, increased stiffness, and ultimately congestive heart disease, this is not sufficient to explain all secondary cardiovascular complications seen in COPD. Instead evidence indicates that intense, non-resolving airway inflammation spilling over from the diseased lung to the heart is a major, yet often overlooked, causative factor behind the clinical association between COPD and heart disease. Taking a more holistic, organ-spanning view of diseases such as COPD can help to stratify patients into those most at risk of cardiac events and to optimise treatment options with a view to improving overall survival by placing more focus on cardiovascular effects as a key outcome for COPD treatments, with different current treatment options capable of both increasing and decreasing risk of cardiovascular events [88].

### ARDS/pneumonia

Acute respiratory distress syndrome (ARDS) is defined as acute hypoxemic respiratory failure with pulmonary edema that is not primarily attributable to cardiogenic lung edema or atelectasis [68] and is most commonly caused by systemic (sepsis) or respiratory (pneumonia) infections. ARDS and pneumonia have long been linked to impaired right ventricular function, which is evident in 22–50% of ARDS patients and has been attributed at large to pulmonary vasoconstriction in response to inflammatory mediators or local atelectasis, and/or to mechanical ventilation with high tidal volumes and peak inspiratory pressures [124]. Yet, recent data suggest that ARDS and pneumonia also commonly trigger acute and chronic cardiac events that are not directly caused by impaired right ventricular hemodynamics but may relate to interorgan crosstalk via immune cells and mediators. Specifically, pneumonia increases the risk for major adverse cardiovascular events including stroke and myocardial infarction to a similar extent as traditional risk factors such as cigarette smoking, diabetes, or hypertension [23]. Conversely, cardiovascular disease increases mortality subsequent to pneumonia, and this risk increases linearly with age [76]. In a recent multicentre study on 1182 patients hospitalized for community-acquired pneumonia, 32% experienced intrahospital cardiovascular events (primarily heart failure, atrial fibrillation, and non-ST elevation myocardial infarction) [118]. Importantly, this increase in cardiovascular risk is not limited to the duration of pneumonia but is still evident in epidemiological studies even 10 years after the patient has been considered fully healed [20]. This persistent risk indicates that cardiovascular events during or subsequent to pneumonia and ARDS are not exclusively attributable to direct effects of the underlying pathogen, but are likely mediated—at least in part—by the host response. The pathomechanisms underlying this complex interdependence between lung infection and inflammation and cardiovascular disease are still largely unclear, as their in-depth interrogation has only recently become feasible with the advent of large-scale unbiased -omics technologies, including single-cell sequencing and high-throughput plasma proteomics, in combination with systems medicine approaches, data science, and artificial intelligence (AI)-based analyses [15]. Several findings point, however, towards a critical role of immune mechanisms as mediators and/or effectors of this disease trajectory from the lung to the cardiovascular system: In non-human primates, severe pneumococcal pneumonia was found to cause acute cardiac toxicity with elevated serum levels of troponin T and ischemic alterations in electrocardiography and echocardiography [91]. Histological evaluation revealed an edematous cardiac interstitium with an increased abundance of small mononuclear cells and neutrophils. In patients with existing atherosclerotic cardiovascular disease, coronary artery calcification—a process closely linked to both systemic and local inflammation [62]—increased more and faster in patients that were hospitalized for pneumonia over a median 5 year follow up relative to patients without pneumonia [21]. Proof-of-principle for a causal relationship between pulmonary inflammation and systemic cardiovascular disease via immune mechanisms was demonstrated in an elegant study by Don Sin’s group, who showed that instillation of the pro-inflammatory bacterial endotoxin lipopolysaccharide into the lungs of atheroprone apolipoprotein E (ApoE)-deficient mice increased the development of vulnerable atherosclerotic plaques by more than fourfold [49]. Importantly, antibody-based depletion of circulating neutrophils attenuated plaque rupture in this model, establishing a direct link between lung inflammation, immune cells, and cardiovascular disease. In line with this notion, recent work by the group of David Dockrell detected a transient increase in macrophage abundance in atherosclerotic plaques following *Streptococcus pneumoniae* infection in ApoE-deficient mice [4]. Increased abundance of macrophages—in particular CCR2^+^ macrophages—was recently also detected by us in cardiac autopsy specimens from patients who died with COVID-19-associated ARDS, and in SARS–CoV–2-infected mice [40]. In the latter model, treatment with a neutralizing antibody against TNF reduced not only the abundance of cardiac monocytes and CCR2^+^ macrophages but also preserved cardiac function, thus establishing a direct mechanistic link between the immune response to pulmonary pathogens and the emergence of cardiovascular disease. Based on the emerging recognition of inflammation-mediated acute and chronic cardiovascular events in pneumonia and ARDS, anti-inflammatory strategies including macrolides, corticosteroids, and statins are increasingly considered adjunctive therapies in this setting [30]. However, definite randomized multicentric clinical trials as to the benefit of these interventions are yet missing, and caution is warranted given the fine balance between prevention of host injury versus impaired pathogen clearance by anti-inflammatory therapies in lung infection.

## Conclusion

Interorgan communication networks are central to health and homeostasis and underpin the proper functioning of physiological systems and pathways. In disease, these same networks can function as conduits for the movement of infectious and inflammatory mediators and activated immune cells from the primary source organ, to secondary sites and expanding, with them, the disease itself. A better understanding of the mechanisms of communication between the heart and lung which enables the expansion of disease into secondary sites is crucial to allow for better management of comorbidities and to optimise survival, as well as helping to identify those patients most at risk of secondary complications of disease.

## Data Availability

No datasets were generated or analysed during the current study.
